# Vitamin D Fortification of Consumption Cow’s Milk: Health, Nutritional and Technological Aspects. A Multidisciplinary Lecture of the Recent Scientific Evidence

**DOI:** 10.3390/molecules26175289

**Published:** 2021-08-31

**Authors:** Luisa Pellegrino, Franca Marangoni, Giovanna Muscogiuri, Paolo D’Incecco, Guillaume T. Duval, Cedric Annweiler, Annamaria Colao

**Affiliations:** 1 Department of Food, Environmental and Nutritional Sciences, University of Milan, Via G. Celoria 2, 20133 Milan, Italy; paolo.dincecco@unimi.it; 2 NFI—Nutrition Foundation of Italy, Viale Tunisia 38, 20124 Milan, Italy; marangoni@nutrition-foundation.it; 3 Department of Clinical Medicine and Surgery, Federico II University of Naples, Via S. Pansini 5, 80131 Naples, Italy; giovanna.muscogiuri@unina.it (G.M.); colao@unina.it (A.C.); 4 UNESCO Chair for Health Education and Sustainable Development, Federico II University of Naples, Via S. Pansini 5, 80131 Naples, Italy; 5 Department of Geriatric Medicine and Memory Clinic, Research Center on Autonomy and Longevity, University Hospital, 49035 Angers, France; duval.guillaume@hotmail.fr (G.T.D.); CeAnnweiler@chu-angers.fr (C.A.); 6 Department of Medical Biophysics, Robarts Research Institute, Schulich School of Medicine and Dentistry, The University of Western Ontario, London, ON N5X 4L2, Canada

**Keywords:** vitamin D, bone health, vitamin D deficiency, milk fortification, consumption milk

## Abstract

Vitamin D is essential in assuring bone health at all stages of life, but its non-skeletal effects are also essential: This vitamin impacts the physiology of the immune system, skeletal muscles and adipose tissue, glucose metabolism, skin, cardiovascular and reproductive systems, neuro-cognitive functions and cell division. The incidence of vitamin D deficiency is widespread worldwide, at any age, in young and healthy subjects, as well as in pregnant women and the elderly population, due to several factors, including inadequate sunlight exposure, skin pigmentation and coverage, adiposity, lifestyle and low dietary intakes. To overcome this problem, the fortification of foods that are consumed on a daily basis, such as milk, is strongly advisable. This opinion paper aims to discuss, in a multidisciplinary way, the current evidence supporting the importance of vitamin D in health and disease and the role of milk as an optimal carrier of this vitamin, to promote adequate intakes, highlighting its unique physico-chemical characteristics linked to both fat globule membrane and casein micelle structure. Moreover, it addresses the impact of industrial processing and storage of consumption milk on the stability of these structures, thus in determining vitamin D bioavailability and the achievement of adequate intakes.

## 1. Introduction

Vitamin D deficiency has become a relevant problem worldwide, at any age: In the elderly population, but also in young and healthy subjects; in fact, the endogenous synthesis of the vitamin may be inadequate even in people living in sunny regions, also due to lifestyle changes occurring in last decades [1,2,3]. Such deficiency, as a consequence, is one of the emerging and most widespread problems of public health, since vitamin D is the main compound responsible for the regulation and control of calcium metabolism and bone health [4,5]. In addition, accumulating evidence describe its role in the regulation of the immune system activity, and specifically in its ability to inhibit the production of inflammatory cytokines and to act in all organs of the human body, contributing to the reduction of the incidence of many common diseases [6,7].

To overcome the limited intakes of vitamin D with common diets, the fortification of foods consumed on a regular basis is frequently adopted to improve the vitamin D status at the population level [8]. Among them, cow’s milk, which represents an important food for a large segment of world population [9], is characterized by high nutrient density at an affordable price [10,11]. In addition, modern technologies for milk sanitation and packaging allow consumption milk to be stored at room temperature up to 10–12 months, thus supplying this food within an increasing trading area. In this context, milk can be a suitable tool to contribute daily to the adequate intake of vitamin D in order to maintain the homeostasis in the blood, reducing the possibility of insufficiency or deficiency. Even if the natural content of vitamin D in cow’s milk is low, i.e., 0.1–1 μg/L in full fat milk [12,13], the fortification of consumption milk with vitamin D has shown to be effective in improving the vitamin intake in many countries [14]. Nowadays, this practice is mandatory in some countries, while in others, is strongly recommended [14].

The aim of this work is to discuss, from a multidisciplinary point of view, the wealth of information currently available on both nutritional and health aspects of vitamin D and the role of cow’s milk as a natural carrier of the vitamin D in human diet.

## 2. Vitamin D

Vitamin D is structurally a steroid-like molecule, with a side chain that is unsaturated in vitamin D2 (ergocalciferol, of vegetable origin) and saturated in vitamin D3 (cholecalciferol, of animal origin); it is biologically inactive and requires double hydroxylation in order to be converted to the active form, able to affect mineral metabolism and to control many other diverse physiological functions [15].

Vitamin D is mostly synthesized in the skin from 7-dehydrocholesterol by ultraviolet irradiation. It binds to an alpha globulin, known as vitamin-D binding protein (DPB), and a small amount is obtained from the diet. It is transported to the liver, where it undergoes the first hydroxylation, and then to the proximal renal tubules, where the second hydroxylation is operated by the 1αhydroxylase, thus resulting in the hormonally active form, namely 1,25-dihydroxyvitamin D (1,25 (OH)2D), which is responsible for most of the biological actions of vitamin D [16].

Probably, due to its widespread effects, 1,25(OH)2D is tightly regulated in its bioavailability and in the processes of activation and deactivation, through a series of negative and positive feedbacks that principally occur in the proximal renal tubule [17]. The negative feedback mechanisms depend on 1,25(OH)2D3 itself, phosphorus, calcium and fibroblast growth factor 23 (FGF-23), whereas the positive ones depend on the parathyroid hormone (PTH), calcium and the type 1 insulin-like growth factor (IGF-1). Low dietary calcium and phosphate result in enhanced activity of 1αhydroxylase. Elevated PTH resulting from hypocalcemia is a primary signal mediating the induction of 1,25(OH)2D synthesis in the kidney. In addition, FGF23 that promotes renal phosphate excretion is also a physiological regulator of vitamin D metabolism. By inhibiting synthesis and promoting catabolism of 1,25(OH)2D, FGF23 functions to reduce the levels of 1,25(OH)2D, which in turn decreases FGF23 expression in bone, forming a negative feedback circuit between FGF23 and the vitamin D endocrine system [18,19].

## 3. Vitamin D and Bone Health

Bone effects of vitamin D have been widely studied. They were actually recognized and explored long before non-bone effects. Vitamin D is involved in bone health at all stages of life, and vitamin D deficiency results in adverse effects on bone structure and global health. It is therefore particularly important to improve overall knowledge of both the prescribers and the population on the bone effects of vitamin D during life span. Vitamin D plays an essential role in the control of bone remodeling through the regulation of phospho-calcium metabolism.

### 3.1. Biomolecular Effects of Vitamin D on Bone

The active metabolite of vitamin D (1,25(OH)2D) stimulates the synthesis of the calcium-binding protein and controls the opening of calcium channels in the intestinal wall leading to an absorption of calcium and phosphate from the intestinal lumen to the bloodstream. These conditions are crucial for bone mineralization [20]. Vitamin D stimulates the activity of bone osteoblasts and increases the production and the plasma concentration of FGF23. Another aspect of the kidney–bone axis is highlighted here involving the active form of vitamin D. FGF23 stimulates the decrease in phosphatemia by urinary excretion and a decrease in circulating PTH. Thus, the bone remodeling activity, which results in bone loss, is reduced [21].

Conversely, in the case of vitamin D deficiency, the absorption of calcium from the digestive tract is reduced, thus reducing the amount of calcium available for bone mineralization. This decrease in serum calcium leads to an increase in PTH (i.e., secondary hyperparathoidism). The increase in serum PTH stimulates the hydroxylation of 25(OH)D to 1,25(OH)2D using the 1αhydroxylase in the proximal tubule of the kidney and promotes bone remodeling, which helps to compensate for the decrease in calcium absorption and restore serum calcium concentration [22]. The stimulation of bone remodeling by PTH during vitamin D deficiency comes at the expense of increased bone loss, which is largely irreversible, and leads to osteoporosis [23]. Exposure to prolonged vitamin D deficiency causes an increased bone turnover, which contains less mineralized osteons and more unmineralized osteoid tissue. The buildup of osteoid tissue associated with prolonged vitamin D deficiency results in osteomalacia [22].

At last, Vitamin D Receptor (VDR) gene polymorphism probably has a responsibility for differences in bone mineral density from one individual to another. Indeed, depending on the allelic variant of the VDR gene and the calcium intake, bone mineral density varies [24].

**Figure 1:**
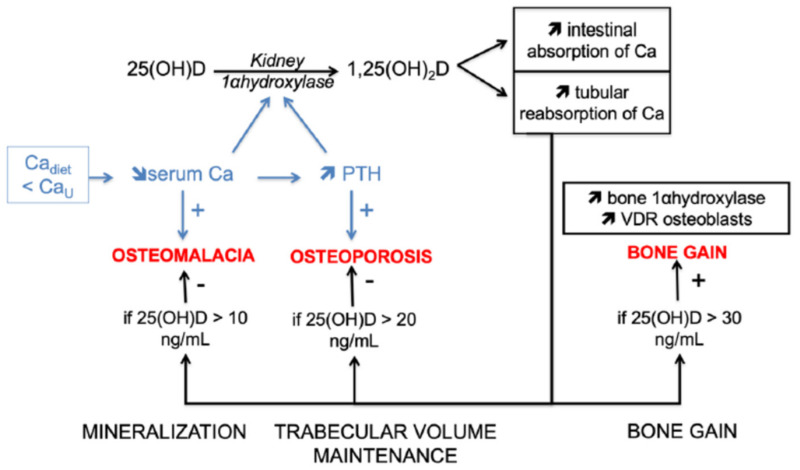
Bone effects of vitamin D according to the different thresholds of 25-hydroxyvitamin D concentration. 1,25(OH)2D: 1,25-dihydroxyvitamin D, 25(OH)D: 25-hydroxyvitamin D, Ca: Calcium, Cadiet: Dietary calcium intake, CaU: Urinary calcium loss, PTH: Parathyroid hormone, +: stimulation, −: Prevention.

There is thus a positive linear relationship between 25(OH)D concentration and bone mineral density (BMD) within physiological limits (i.e., 25(OH)D < 100–120 ng/mL). In practice, there are two critical thresholds to fully understand this relationship (Figure 1): A threshold of “calcium homeostasis” corresponding to the stimulation of the activity of kidney 1αhydroxylase, when the concentration of 25(OH)D is above 10 ng/mL (mineralization action), and a threshold of “bone homeostasis” corresponding to the stimulation of the activity of bone 1αhydroxylase and to the increase in the expression of bone VDRs when the concentration of 25(OH)D is above 30 ng/mL (bone gain action). The intermediate concentration of 25(OH)D, around 20 ng/mL, helps prevent secondary hyperparathyroidism and therefore maintains trabecular volume.

### 3.2. Clinical Effects of Vitamin D Deficiency on Bone According to Age

(a) Infants and young children

The role of vitamin D in building bone in fetuses and newborns has long been known. The first cases of rickets described date from the 17th century. In fact, rickets is a disease of bone growth and mineralization resulting from vitamin D and calcium deficiency during the bone development period of infants and young children [25]. Vitamin D deficiency in this population is mainly linked to a lack of sun exposure and a low vitamin D diet. Different socio-geographic factors can lead to vitamin D deficiency in pregnant women, infants and young children. Clinical consequences of rickets are bone deformations affecting the entire skeleton but mainly affecting long bones and metaphysis growth cartilages. These abnormalities can lead to dwarfism in some extreme cases.

(b) Adults

In adults, clinical signs suggestive of a vitamin D deficiency are mainly bone discomfort or pain that is generalized or often localized in the pelvis, the lumbar region and the lower limbs, impaired physical function, muscle pain and muscle fatigue [26,27].

(c) Elderly people

The prevalence of vitamin D deficiency is very high, up to 90%, in older adults [28]. Elderly people are more at risk of vitamin D deficiency due to a frequent decrease in sun exposure, a decreased absorption of vitamin D from food and a decrease of skin vitamin D synthesis and kidney hydroxylation [29]. Vitamin D deficiency leads to secondary hyperparathyroidism, which is responsible for bone loss. This bone loss can lead to mineralization defect, osteoporosis and osteomalacia in the case of prolonged exposure. Vitamin D deficiency also exposes one to a risk of fractures due to bone fragility and an increased risk of muscle fatigability [30] and falls [31].

### 3.3. Bone Effects of Vitamin D Supplementation According to Age

The administration of vitamin D supplements, to prevent or control symptoms of deficiency, has a role in conditions that can be found practically at any age.

(a) Infants and young children

Vitamin D supplementation in infants and young children is mainly to prevent rickets. Before the development of synthetic vitamin D, the consumption of cod liver oil rich in vitamin D was recommended. Daily vitamin D supplementation is usually proposed in the last trimester of pregnancy for pregnant women and for newborns from birth to 18 months of age. Thereafter, more spaced treatment, often in the winter months, is proposed until the end of bone growth. This last point depends on risk factors and socio-geographic status. It is also interesting to note that exclusive breastfeeding is at greater risk of vitamin D deficiency than when using formula milk very often supplemented with vitamin D.

According to the Scientific Advisory Committee on Nutrition (SACN) and the National Institute for health and Care Excellence (NICE) Guidelines, a reference nutrient intake (RNI) of 8.5 to 10 µg (15.2 to 17.8 nanomoles) per day is recommended for all infants from birth to 1 year of age, and 10 µg (17.8 nanomoles) per day for children aged 1 to 4 years [7,26]. Nutritional intake of vitamin D refers to the consumption of foods naturally rich in vitamin D, fortified products such as growth milk and supplementation.

Higher daily doses of vitamin D or loading doses are given transitorily in the treatment of rickets. A maintenance treatment is then implemented as a follow-up to the curative treatment. Calcium supplementation may also be proposed. Orthopedic surgery is sometimes necessary, especially for severe genu valgum and genu varum causing locomotor disorders [32].

(b) Adults

In healthy middle-aged adults, reference nutrient intakes are established to ensure a sufficient vitamin D concentration for most of the population in order to avoid the harmful musculoskeletal effects of vitamin D deficiency. These recommendations of 10 micrograms (17.8 nanomoles) per day for adults are also applicable to a larger population aged 4 years and older. Reference nutrient intakes are the same for pregnant and lactating women and population groups at increased risk of vitamin D deficiency. However, these guidelines do not consider sun exposure, season, latitude and complex parameters affecting the skin synthesis of vitamin D [7,26]. In this population subgroup, vitamin D drug supplementation should not be universal but implemented only in individuals with vitamin D deficiency. Varied diet and outdoor activities should be encouraged.

(c) Elderly people

In the older population, blood testing is recommended in those at risk of or exhibiting suggestive symptoms of vitamin D deficiency or insufficiency [7]: Osteoporosis, rickets, osteomalacia, fragility fracture, osteoporosis inducing treatment or repeated falls. Other guidelines also recommend to systematically supplement all residents in nursing-homes [33].

Vitamin D supplementation in this population reduces the risks of osteomalacia, osteoporosis and fractures. Oral vitamin D supplementation between 700 and 800 UI/day is necessary to reduce the risk of hip and other non-vertebral fractures [34]. This preventive effect is dose dependent [35] and found in those with vitamin D deficiency at baseline [36]. In a meta-analysis of 11 observational studies (39,141 participants, 6278 fractures, 2367 hip fractures), each increase of 10.0 ng/mL (i.e., 25 nmol/L) in 25(OH)D concentration was associated with an adjusted relative risk (RR) for any fracture of 0.93 (95% CI, 0.89–0.96) and an adjusted RR for hip fracture of 0.80 (95% CI, 0.75–0.86) [37]. The combination of vitamin D with calcium may be more efficient than vitamin D alone since the latter meta-analysis also found that daily supplementation with both vitamin D and calcium (6 randomized clinical trials with 49,282 participants, 5449 fractures, 730 hip fractures) was associated with a 6% reduced risk of any fracture (RR, 0.94; 95% CI, 0.89–0.99) and a 16% reduced risk of hip fracture (RR, 0.84; 95% CI, 0.72–0.97) [37]. However, it is noticeable that vitamin D supplementation exhibits only a low-amplitude effect on the bone mineral density of 0–2%, which may not explain the prevention of fractures [38], the latter one being likely explained by the prevention of falls in older people [35,36].

## 4. Vitamin D and Non-Skeletal Effects

Although the role of vitamin D in calcium-phosphorus metabolism and in the homeostasis of bone mineral reserves is the most well-known, its effects on the homeostasis of non-skeletal tissues are equally documented. VDR are in fact ubiquitous, being widely ex-pressed in all tissues, including breast, endothelium, vascular smooth muscle, cardiomyocytes and the male urogenital system. Indeed, it has been observed that vitamin D plays a fundamental role in the physiology of the immune system, skeletal muscles and adipose tissue, glucose metabolism, skin, cardiovascular and reproductive systems, neuro-cognitive functions and cell division and sleep regulation [39,40,41].

The role of vitamin D on the immune system has been the subject of a breadth of literature [42] and of a scientific opinion of the European Food Safety Authority (EFSA), which approved the use of the following claim: “Vitamin D contributes to the normal function of the immune system and healthy inflammatory response” for foods which contain significant amounts of the vitamin [6].

All the cells of the immune system, as far as it is known to date, express VDR. The cell presenting antigen (APC), upon immune stimuli, are capable of producing 1,25(OH) 2D3 through the same enzyme expressed in the kidney. 1,25(OH)2D3 exerts its action on both the innate and the acquired immunity. On innate immunity, vitamin D and its metabolites stimulate the differentiation and activation of macrophages, producing defensins, such as cathelicidin and β2-defensin [43]. The main effects of vitamin D on acquired immunity are inhibitory, causing a phenotypic shift of T cells from an effector phenotype, involved in autoimmune diseases, to a regulatory and protective one.

## 5. Vitamin D Status: Role of the Dietary Intake

### 5.1. Epidemiology

Available data suggest that vitamin D deficiency is widespread worldwide, with the highest prevalence in Asia, the Middle East and Africa, as well as among immigrants from these regions living in countries at higher latitudes [44]. Even if data regarding vitamin D status were mainly from studies on selected small samples, there is consistent evidence suggesting that the risk of vitamin D deficiency would affect a larger part of general population, as shown by the ODIN project [45], funded by the European Commission. The map of vitamin D status in different countries (from 107 studies involving more than 630,000 subjects) shows that, overall, one person in eight is at risk of vitamin D deficiency, even in sunny regions [1].

The overall pooled estimate, irrespective of age group, ethnic mix and latitude registered serum 25(OH)D concentrations <12 ng/mL in 13.0% of the total European population on average in the year, and specifically in 17.7% of individuals sampled during the extended winter period (October–March) and 8.3% in those sampled in summer (April–November). In the same population, the mean prevalence rises to 40.4% if considering an alternate suggested definition of vitamin D deficiency (<20 ng/mL) [1].

This observation is confirmed by another survey by the International Osteoporosis Foundation: Circulating levels of vitamin D are, on average, below 20 ng/mL, i.e., in the range considered ‘insufficient’, also in populations living in the Mediterranean area [46].

Outside Europe, the risk of vitamin D deficiency has also been described in the Middle East, China, Mongolia and India, in risk groups including older persons and pregnant women. Adequate vitamin D status, defined as serum 25-hydroxyvitamin D greater than 20 ng/mL, is present in less than 50% of the world population, at least in winter [3]. Within a nationally representative sample of Australian adults aged ≥25 years (the 2011–2013 Australian Health Survey, n 5034), 20% of participants (19% of men and 21% of women) were classified as vitamin D deficient (serum 25(OH)D concentrations <20 ng/mL), with a further 43% (45% of men and 42% of women) classified as insufficient (Vitamin D: 20 to 30 ng/mL).

In the US, almost one-fifth (18.3%) of the NHANES 2011–2014 population (>1 y to >60 y) had serum 25(OH)D values categorized as at risk of inadequacy (12–20 ng/mL) and 5.0% were at risk of deficiency (<12 ng/mL) [2]. In particular, among adults aged 20–39, 7.6% were at risk of deficiency and 23.8% at risk of inadequacy; the prevalence is slightly lower among people aged 40–59 (5.7% and 18.6% at risk of deficiency and inadequacy respectively). Vitamin D deficiency has been reported to increase in the elderly, not only due to reduced skin production of vitamin D with age, but also because of age-related factors that can result in limited sun exposure, such as being more housebound [47]. In fact, some studies have shown that the risk of vitamin D deficiency increases up to 80% in institutionalized elderly [48]. A high prevalence of vitamin D deficiency has been described even among pregnant women in the Mediterranean regions (50–65% in most studies), with a certain variability between different European countries, resulting in severe skeletal and non-skeletal health events among offspring [49]. A similar prevalence has been described in the US on average, with great differences between white (13%), Hispanic (45%) and black (80%) pregnant women. As regards children and adolescents, for some years now pediatricians have been pointing out that vitamin D deficiency occurs commonly among these population groups, even in Europe. Limited data on vitamin D concentrations and vitamin D deficiency among the healthy pediatric population are available from several countries, including Denmark, England, Finland, France, Germany, Greece, Ireland, Italy, the Netherlands, Poland, Spain, Switzerland and Turkey [50]. Although some of the studies included only small numbers of children, they allow to conclude that the main causes are obesity, dark skin and inadequate sun exposure due to living in northern latitudes, excessive use of sunscreen with high SPF, staying indoors for much of the day and wearing clothes covering most of the skin [50]. More systematic information has been collected in the US where, according to NHANES 2011–2014 data, 6.6% of children aged 1–5, 12.3% of children aged 6–11 and 22.7% of adolescents (12–19 y) are at risk of inadequacy [51]. The increase in prevalence of vitamin D inadequacy (and deficiency) throughout the various age groups has been confirmed in Great Britain: According to data from the National Diet and Nutrition Survey (NDNS) (1102 samples) vitamin D circulating levels decreased progressively with age in both sexes, moving from 30 ng/mL (4–8 y) to 25.2 (9–13 y) to 16.5 (14–18 y) on average [52].

### 5.2. Nutritional Recommendations and Dietary Intake

The role of vitamin D intake through the diet in cases of inadequate exposure to sunlight has been emphasized by the EFSA, which has recognized an evidence-based direct association between the intake of significant amounts of the vitamin and some important health effects for the general population [4]: Both at the musculoskeletal level—by contributing to normal absorption/use of calcium and phosphorus, normal blood calcium levels, maintenance of normal bones and teeth and normal muscle function—and on the immune system, by contributing to its normal function (in infants and children too). The corresponding health claims are therefore authorized by the European Commission for products that are at least the “source” of the vitamin, i.e., containing at least 15% per 100 g or 100 mL or per consumption unit, of the nutrient reference value set at 5 μg/day [53].

The tolerable upper intake level of vitamin D has been set at 100 µg/day (4000 IU) by both EFSA in 2012 and the Institute of Medicine (IOM) in 2011 for adolescents and adults, including pregnant and lactating women [5,54]. Lower tolerable levels have been defined for infants (EFSA: 25 µg/day; IOM: 62.5 µg/day) and for children (EFSA: 50 µg/day for 1–10 years; IOM: 75 µg/day for 1–8 years).

The reference value is slightly lower than the intake of vitamin D that is considered adequate to maintain normal serum levels of 25(OH)D when skin synthesis is minimal, which is set, in Europe, at 10 μg/day for children 7–11 months of age, the same as in the United States, and to 15 μg/day for children aged 1–17 years and adults (including pregnant and lactating women), to allow the majority of the population to achieve a serum 25(OH)D concentration near or above the target of 50 nmol/L [55]. Because of lack of sun exposure and the decline with age of endogenous vitamin D synthesis, a higher reference intake, i.e., 20 μg/day, is set for people over 65 years of age, by the Institute of Medicine, the Health Council of the Netherlands, the Italian Society of Human Nutrition and the Nordic Council of Ministers [5,55]. Requirements of food-derived vitamin D may be lower with regular exposure to UVB-containing sunlight and when skin synthesis is adequate [44].

However, dietary sources of vitamin D are limited: The best available natural source is cod liver oil (90–250 μg/100g), followed by fatty fish (e.g., 6–10 μg/100 g of aquaculture salmon); lower concentrations are in lean fish, eggs, meat and dairy products [56]. Fortified foods make the highest contribution to vitamin D intakes in countries where fortification is recommended or mandatory; in the US, vitamin D-fortified milk makes the highest contribution to vitamin D intakes (58% in men, 39% in women), while in other countries without fortification policies, the supply of vitamin D with milk is quite low [47].

**Figure 2:**
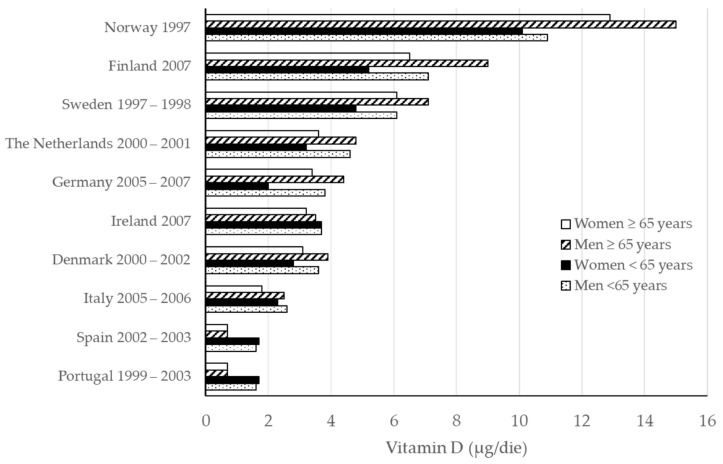
Vitamin D intake (μg/day) in adults living in some European countries (modified from [47]).

Generally speaking, the results of surveys on representative samples of the general population in Europe suggest that dietary vitamin D intake is low overall and, on average, far from even the 5 μg set as a daily reference value. The EPIC (European Prospective Investigation into Cancer and Nutrition) study showed that vitamin D content is different in diets of people living in the 10 countries involved in the project, with intakes below mean values recorded among men and women recruited in Italy (over half of the European mean intake) and France, and intakes above the EPIC mean in Scandinavian countries (Norway and Sweden) [57]. A similar observation, namely the geographical gradient of dietary vitamin D from Northern to Southern Europe, has been described by other authors and also for children [47,58] (Figure 2). This variability can be explained by the higher consumption in Nordic countries of food products that are frequently fortified with vitamin D, where they play an important role in enabling the general population to meet the vitamin requirement.

In particular, milk is among the foods that are most commonly supplemented with vitamin D, to enhance the adequate intake of the vitamin in the general population, being of common and daily consumption for a large part of any population around the world [59].

In fact, cow’s milk (and dairy products in general) has been part of the human diet for millennia, being characterized by a favorable nutrient profile; it is composed of about 87% water and also contains, on average, 3.5% protein, about 5% lactose, an amount of fat variable from 0.5% (in skimmed milk) to 3–4% (in whole milk) and minerals (1.2% mainly calcium and phosphorus) [60]. However, the content of liposoluble vitamins is low and that of vitamin D can be negligible in raw milk, as well as in yogurt [12].

## 6. Milk as an Optimal Carrier of Vitamin D

Milk is a tremendously complex system from a physico-chemical point of view. The bio-synthetic processes involved in milk secretion require the mammary cells to perform an uninterrupted work of filtration of precursors and water from blood stream [61]. It has been estimated that more than 500 L of blood have to flow through the udder to produce one liter of milk. Within the mammary epithelial cells, precursors are constantly assembled into milk components, some of which have to be further organized in specific supramolecular structures with a high degree of complexity, i.e., casein micelles and fat globules. It has been recently suggested that these structures are envisioned in milk to act as natural nanocarriers of biologically active molecules that can thus be delivered to the neonate [62]. The comprehension of this intriguing perspective needs a discussion in light of the most recent acquisitions regarding selected properties of protein and fat structures that make them suitable to play that role.

### 6.1. Casein Micelles and Whey Proteins

**Figure 3:**
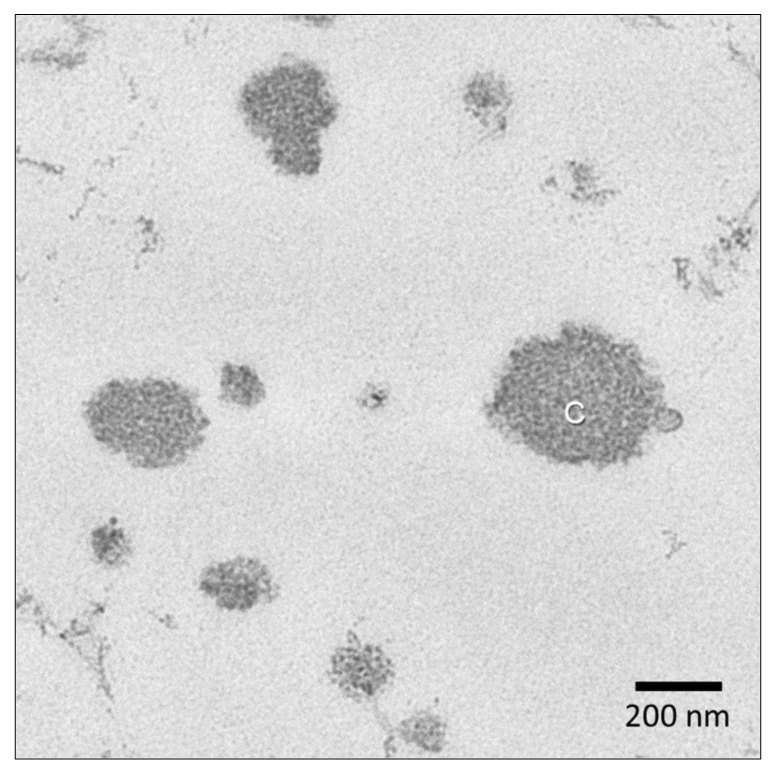
Transmission electron microscopy of casein micelles (C) in raw milk. Sample was prepared as described by D’Incecco et al. 2018 [66].

Milk proteins comprise two main groups: Casein and whey proteins. Casein represents 80% of milk proteins and is present in a colloidal form, namely micelles, that gives the milk the white appearance. Around 10^14^–10^16^ micelles are present in one milliliter of milk. Despite the number of studies carried out even in recent years, the structure of casein micelles is still partly unknown. Casein is actually a family of four distinct proteins, i.e., β-, αs1-, αs2- and κ-casein, with a scarcely organized structure and a strong tendency to self-associate into micelles through interactions of hydrophobic segments in the protein chains, hydrogen bonds, ionic bonds and van der Waals interactions. This architecture is stabilized by nanoclusters of calcium phosphate embedded within the protein matrix. Furthermore, the less hydrophobic κ-casein molecules are mainly positioned on the periphery of the micelle and partly extend outward forming a strongly hydrated brush layer on the surface. The overall features of casein micelles have been largely consolidated by research studies conducted over decades. Microscopy techniques such as transmission electron microscopy give evidence of a roughly spherical shape (Figure 3), and a size distribution roughly ranging from 50 to 500 nm is confirmed by light scattering measurements. How-ever, several aspects have not yet been fully elucidated. Among these, the relative distribution of water and protein throughout the micelle is still under debate. Micelles are highly hydrated, with 3–3.5 g water per g protein, and 55–60% of water associated with the micelle is entrapped in the inner part [63]. Recent studies assign the casein micelle a sponge-like structure, with many pores on the surface and channels in the core portion. The presence of numerous cavities 20–30 nm wide has been observed, filled of water and interconnected by irregular channels of about 5 nm in diameter [64]. While functional and technological behavior of casein typically depends on the surface properties of the micelles and the environmental conditions (pH, temperature, ionic concentration), the inner structural features are likely relevant in allowing the casein micelle to act as a carrier of bioactive compounds. In particular, it is emerging that lipophilic bioactives, like vitamin D, can be carried also by casein micelles besides fat globules [65]. Owing to the sponge-like structure of the micelle, these compounds can be involved in dynamic exchanges, i.e., retention into the micelle and release, depending on their characteristics.

Globular proteins such as milk whey proteins also have the capability of carrying hydrophobic compounds by surface interaction, but the ligand can also enter the cavity of the protein. The native β-lactoglobulin is a good carrier of vitamin D, retinol and fatty acids [67] (pp. 211–259), whereas α-lactalbumin has a specific site for binding vitamin D [68]. Blood serum albumin binds vitamins, hormones and nucleotides. Furthermore, bovine milk contains the vitamin-D binding protein, a specific carrier glycoprotein with molecular characteristics close to the homologous protein in human milk [69].

### 6.2. Fat Globules

**Figure 4:**
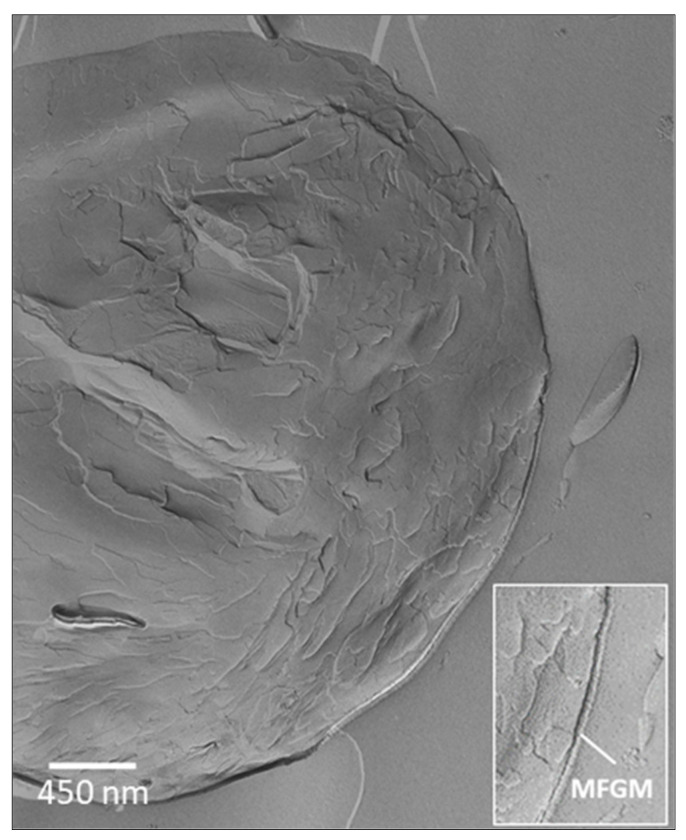
Freeze-fracturing transmission electron microscopy of a milk fat globule in raw milk and (box) milk fat globule membrane (MFGM).

Fat globules are lipid droplets 0.1–10 µm in size, naturally occurring in milk as a fine emulsion. Fat globules are surrounded by a complex membrane, 8–10 nm thick, comprised of phospholipids, proteins, sterols and enzymes [70]. The membrane prevents fat globule coalescence and flocculation, thus providing stability to the emulsion phase [71]. The lipid core of the globule is primarily a mixture of triglycerides, but it also comprises a variety of lipid-soluble compounds that are entrapped together with the fat during globule assembling in the mammary cell. The lamellar distribution of triglycerides within the globule as well as the globule membrane are well visible by transmission electron microscopy (Figure 4). The presence of compounds such as vitamin D, retinol (vitamin A) and β-carotene contributes to protect milk fat from oxidation [62]. It has been demonstrated that these compounds move through the fat globule membrane by a passive diffusion process. Thus, an additional amount of vitamin D can be loaded into the native fat globules by permeation to the lipid core through the membrane [72] that, in turn, protects the vitamin D against the acidic conditions of the gastric environment. Studies on gastrointestinal digestion of milk indicate that the presence of membrane fragments in the gut lumen promotes the interaction with bile [73].

### 6.3. Industrial Processes and Storage of Milk

The impact of industrial processing and storage of consumption milk on the stability of vitamin D determines the actual intake of this compound. Overall, it has been reported that vitamin D3 in dairy products is more stable than the other forms [8]. While vitamin D itself is prone to degradation by heat, light and oxygen, it becomes very stable when protected by food matrix. Differently, the processing and storage conditions induce deep changes to milk components, namely protein and fat.

Raw milk refrigeration is a common practice during transportation and at the processing plants. However, storage at low temperature has been shown to result in slow release of phospholipids from the fat globule membrane that becomes progressively thinner and weaker [70]. A similar structure weakening involves casein micelles due to the cold-induced release of β-casein and calcium phosphate.

**Figure 5:**
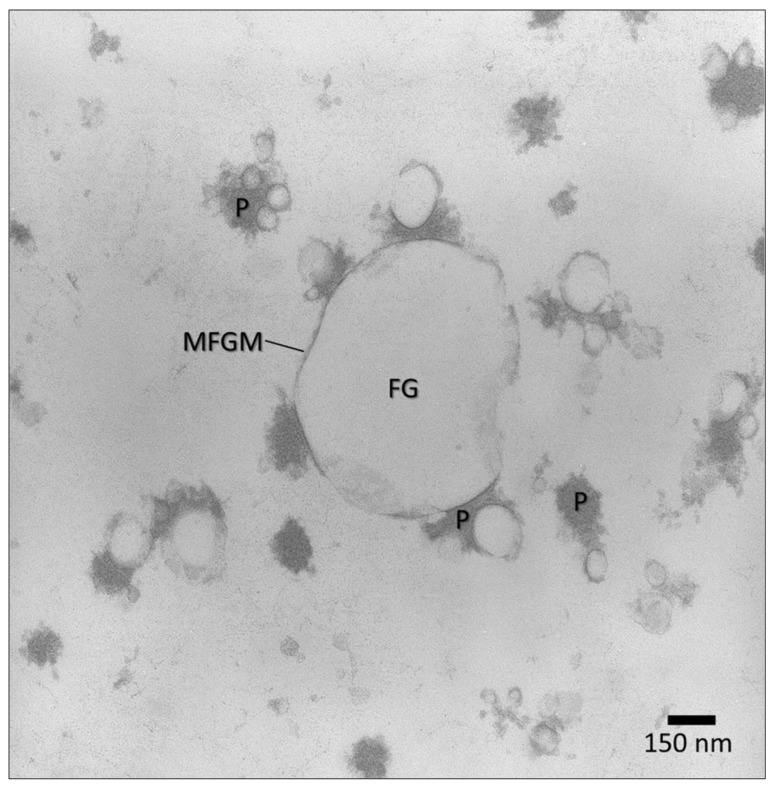
Transmission electron microscopy of fat globules (FG) and protein (P) in homogenized pasteurized milk. The sample was prepared as described by D’Incecco et al. 2018 [66]. MFGM = milk fat globule membrane.

Homogenization is also a common step in the industrial manufacture of drinking milk. Basically, it is a high-pressure treatment aimed at mechanically disrupting the fat globules into smaller (<1 µm) particles that are more stable against creaming. The surface of newly formed globules may increase up to 20–30 times [74] and thus casein micelles and whey protein molecules are adsorbed at the surface to rebuild an interface between the fat core and the aqueous environment outside [66] (Figure 5). Though very important for fat digestion, milk homogenization alters both the permeability of the membrane and the surface activity of fat globules compared to the native status.

Thermal treatments are used to destroy spoilage microorganisms, including any pathogens, and to preserve milk quality during an adequate shelf-life period. Vitamin D is known to be stable to thermal treatments commercially adopted by the dairy industry, i.e., pasteurization and UHT sterilization [75]. In contrast, changes induced by heating milk components, like those induced by homogenization, have an indirect effect on their capability to act as a carrier of micronutrients including vitamins. Depending on the process intensity, progressively more whey proteins are denatured and irreversibly interact with both casein micelles and proteins of fat globule membrane. It should also be mentioned that the current health trends promote the consumption of low-fat dairy products. In these products, mostly proteins act as nanocarriers for vitamin D and other hydrophobic nutraceuticals. Due to the increasing demand for protein-based nanocarriers for the delivery of these compounds, structures have been developed that mimic the behavior of native casein micelles [65].

## 7. Fortification Policies

All the described characteristics of milk, making it an optimal carrier for vitamin D, underlie the decision of some governments to encourage, or in some cases to compel, companies producing specific categories of foods to add vitamin D to a significant extent, on the basis of a solid nutritional rationale [14]. This is the case of foods, commonly containing very low amounts of the vitamin, that are consumed regularly and on a daily basis, which can provide, if fortified, a steady and nutritionally relevant supply of the vitamin [76]. In particular, the fortification of milk (as well as spreadable fats) is strongly recommended in Finland (1 µg/100 g in milk, yogurt and sour milk), while in Sweden, fortification is mandatory for milk with a fat content below 3% by weight (as well as for vegetable drinks intended for use as alternatives to milk: 0.38–0.50 µg/100 g). A similar approach has been adopted by Canada (milk: 0.825–1.125 µg/100 g) [14].

The safety of consumption of vitamin D-fortified foods has been confirmed by several studies [77]. Data from surveys carried out in 14 European countries indicate that vitamin D intakes from all sources, including fortified foods, within commonly consumed diets, are far below the upper tolerable levels for both adults and children [54].

The effectiveness of policies aimed at improving intakes of vitamin D by the general population through food fortification, whose adoption is relatively recent, has begun to be reported in recent years. In Canada, the fortification of milk, yogurt and cheese at 6.75 μg (270 IU)/serving led to more than doubling the vitamin D intakes across all sex/age groups and a drop in the prevalence of dietary inadequacy, from >80% to <50%, without a risk of excess [78]. Similarly, the improvement of vitamin D status in the Finnish adult population between 2000 and 2011 has been mainly explained by food fortification, especially of fluid milk products (whose contribution to dietary vitamin D intake changed from 4 to 34% in the same period), other than augmented vitamin D supplement use [79]. At the same time, the risk of insufficient vitamin D levels was reduced in population groups consuming fortified milk on a regular basis in comparison with those excluding this food from the diet, of both adults and children: Milk fortified with vitamin D was the main dietary source of vitamin D, providing 48.7% of daily intake in a population of Finnish children [80].

The study of other health effects of policies aimed at improving vitamin D intakes through food fortification is still ongoing. For example, in Denmark, prenatal exposure to small amounts of extra vitamin D from food fortification has been associated with a lower risk of developing inflammatory bowel disease before 30 years of age [81].

## 8. Conclusions

The critical evaluation of the relevant literature on clinical, nutritional and technological aspects allows to highlight the following points: (1) Vitamin D is essential in bone remodeling throughout the lifespan, with some specificities at each age, and for the homeostasis of many other essential metabolic processes; (2) vitamin D deficiency, which is widespread worldwide, potentially causes serious adverse effects such as rickets, osteomalacia or osteoporosis with a high risk of bone fractures and impacts on the functionality and maintenance of many physiological systems; (3) the risk of vitamin D deficiency has been described in different population groups, even in people living in sunny regions; (4) the contribution of the diet to the daily amount of vitamin D is overall modest, in the absence of fortified foods; (5) the fortification of foods consumed on a daily basis has shown to be effective in improving the intake of the vitamin in the general population; (6) in particular, the fortification of cow’s milk with vitamin D is strongly recommended by some governments; (7) the assessment of the unique compositional characteristics of milk allows to conclude that it represents a good carrier for vitamin D, able to provide a small but significant amount of vitamin D, also contributing to reaching the adequate daily intake.
